# Widely Targeted Metabolomic Analysis of Two Chinese Traditional Herbal Imperial Chrysanthemum Teas and In Vitro Evaluation of Their Hyperglycemia and Inflammation Enzyme Inhibitory Activities

**DOI:** 10.3390/foods14173142

**Published:** 2025-09-08

**Authors:** Yang Liu, Di Wang, Liqing Mei, Jiaying Liang, Yuqin Xu, Jie Teng

**Affiliations:** College of Agriculture, Jiangxi Agricultural University, Nanchang 330045, China; liuyang@jxau.edu.cn (Y.L.);

**Keywords:** imperial chrysanthemum tea, widely targeted metabolomics, flavonoids, biological activities

## Abstract

Imperial chrysanthemum teas ‘Wuyuan Huangju’ (WYHJ) and ‘Jinsi Huangju’ (JSHJ), dried from the flowers of *Chrysanthemum morifolium* cv. Huangju, are traditional and popular herbal teas in China. However, their metabolite profiles and bioactivities remain unclear. In this study, we aimed to comprehensively elucidate the non-volatile and volatile metabolites of these two imperial chrysanthemum teas and assess their antioxidant activities and inhibitory effects on hyperglycemia and inflammation enzymes. Thus, we employed a widely targeted metabolomics approach based on UPLC-ESI-MS/MS and GC-MS/MS to characterize metabolite profiles of the two teas. In total, 1971 non-volatile and 1039 volatile metabolites were explored, and among these, 744 differential non-volatiles (classified into 11 categories) and 517 differential volatiles (classified into 12 categories) were identified. Further, 474 differential non-volatiles were upregulated in WYHJ, particularly flavonoids, terpenoids, and phenolic acids. In contrast, JSHJ exhibited a greater number of upregulated differential volatiles compared to WYHJ, contributing primarily to its sweet, fruity, and floral aroma. The results of scavenging activities towards DPPH·, ABTS·^+^, OH·^−^, and reducing power demonstrated that both imperial chrysanthemum teas, especially WYHJ, displayed high antioxidant capacity. We also noted that WYHJ exhibited stronger α-amylase, α-glucosidase, xanthine oxidase, and lipoxygenase inhibitory effects owing to its high active substance content. Therefore, this study provides insights into the metabolites of Chinese traditional medicinal herbal teas and highlights strategies for the comprehensive development and utilization of these traditional plant resources.

## 1. Introduction

*Chrysanthemum morifolium* cv. Huangju, also known as imperial chrysanthemum, is a Chinese plant widely used in teas and beverages, occupying an important position in the edible flower market [1,2]. Imperial chrysanthemum tea, derived from the dried anthodium of *Chrysanthemum morifolium* cv. Huangju, has also become a popular herbal tea in China owing to its beneficial health effects, appealing appearance, and unique flavor [3]. Specifically, Wuyuan Huangju (WYHJ) and Jinsi Huangju (JSHJ), which differ in appearance and quality, are the two main imperial chrysanthemum tea varieties [4]. However, there is limited comparative information available regarding their morphologies, constituents, and physiological activities. To further promote the use of these two imperial chrysanthemum teas in functional beverages and foods, a scientific description and comparative study are necessary.

Chrysanthemum plants and their associated taxa have traditionally been utilized as ethnomedicines and are recognized for their numerous health benefits, including protection against oxidative damage, inhibition of inflammation, and improvement of hyperglycemia and eye health [5]. A large body of evidence suggests that flavonoids (e.g., Lut-7-O-glucoside, Acn-7-O-rutinoside, gallocatechin, anthocyanins) and phenolic acids (e.g., caffeoylquinic and chlorogenic acids) in chrysanthemum have strong anti-inflammatory properties [6,7]. Further, in recent decades, the key enzyme inhibition theory has emerged as an explanation for the efficacy of some plant components in exerting multiple health benefits [8]. Reportedly, the activities of α-amylase and α-glucosidase are associated with hyperglycaemia, while xanthine oxidase (XOD) and lipoxygenase (LOX) play pivotal roles in triggering inflammation via the gout and arachidonic acid pathways, respectively [9,10]. The key enzyme inhibition potential of the metabolites in some *Chrysanthemum* species has been reported. For example, flavonoids in *C. indicum* regulate postprandial glucose levels by inhibiting α-amylase activity [11]. However, studies on the physiological effects of imperial chrysanthemum tea components remain limited, highlighting an urgent need to explore the phytometabolites in the two imperial chrysanthemum teas. Therefore, we hypothesize that the two imperial chrysanthemum teas exhibit physiological activities similar to those of other *Chrysanthemum* teas.

Phytometabolites identification is important for evaluating the physiological activities of plants and can effectively guide the thorough exploration and utilization of medicinal plant resources [7]. The significant differences in metabolites between different *Chrysanthemum* tea varieties have been extensively reported [12]. However, the metabolite profile of imperial chrysanthemum teas remains unclear. A study analyzing 17 commercial chrysanthemum teas revealed differences in chemical compositions among the samples, indicating that their distinct compositions lead to varying anti-inflammatory activities in vitro [13]. Furthermore, volatile content is a critical parameter that determines the commercial quality of chrysanthemum teas. Partial least squares discriminant analysis has demonstrated that chrysanthemum teas, including chamomile, ‘Xiaokuixiang’, ‘Hangju’, and ‘Huangju’ contain various volatile components [14]. Most studies on imperial chrysanthemum tea metabolites have focused on the comparison and analysis of a specific tea as a reference variety [15,16]. Comprehensive studies exploring the differences between similar varieties are limited. Therefore, this study comprehensively investigates and compares two imperial chrysanthemum tea varieties, focusing on their morphologies as well as their non-volatile and volatile metabolites.

Widely targeted metabolomics is a high-throughput technique that offers broad coverage and shows high sensitivity [17]. It is a powerful tool for analyzing and characterizing the quality and composition of foods. This innovative technology holds significant potential for the comprehensive detection and analysis of metabolites in imperial chrysanthemum tea. In this study, we conducted a thorough metabolomics profiling of both non-volatile and volatile metabolites of imperial chrysanthemum tea using UPLC-ESI-MS/MS and GC-MS/MS. Subsequently, we examined their antioxidant activities and the effects of these metabolites on enzymes related to hyperglycemia and inflammation. Therefore, the finding of this study may provide valuable insights into the functional chemical composition of traditional Chinese herbal teas.

## 2. Materials and Methods

### 2.1. Material Collection and Chemical Reagents

Fresh *Chrysanthemum morifolium* cv. Huangju flowers were supplied by Wuyuan Huayuan Tea Products & Services Co. Ltd. (Wuyuan, Jiangxi Province, China (29°25′40″ N; 117°86′84″ E)) in November 2023. The plants were cultivated under standard agronomic conditions without the use of pesticides. Flowers were selected at a uniform, fully open stage based on intact morphology, and the absence of physical damage or pests. A total of three independent biological batches (approximately 1 kg fresh weight each) were collected from different sub-plots within the plantation to account for biological variability. To obtain the two imperial chrysanthemum teas, the fresh flowers were dried with the temperature gradually increasing from 30 to 80 °C until their water content decreased to less than 5%. The dried flowers were then immediately stored at −80 °C until further analysis.

Methanol, acetonitrile, hexane, and formic acid were purchased from Merck (Darmstadt, Germany). Xanthine, acarbose, linoleic acid, sodium phosphate (PBS), sodium chloride, 4-nitrophenyl α-D-galactopyranoside (PNPG), ascorbic acid (V_C_), allopurinol, nordihydroguaiaretic acid (NDGA), and dinitrosalicylic acid (DNS) were purchased from Adamas (Shanghai, China).

### 2.2. Flower Morphology Analysis

The main morphological characteristics of the fresh imperial chrysanthemum flowers, including weight, diameter, length, and number of ligulate flowers, were explored. To determine the weight of a single flower, ten fresh flowers of each species were weighed on an electronic balance, and the average weight was calculated. The diameter of 10 flowers of each species were determined by measuring the widest parts of the flowers using a Vernier caliper. Thereafter, the average diameters were calculated. Further, the length and number of ligulate flowers were determined by measuring 10 samples for each species and thereafter, calculating the respective average values.

### 2.3. Analysis of Non-Volatile Metabolites in Imperial Chrysanthemum Teas

#### 2.3.1. Sample Preparation

Dried imperial chrysanthemum tea samples were first ground into powder. Thereafter, 50 mg powder samples were accurately weighed and mixed with 1.2 mL of pre-cooled 70% methanol aqueous solution. Centrifugation was then performed at 1000*× g* for 3 min and the resulting supernatant was aspirated, filtered through a 0.22 μm microporous filter membrane (SCAA-104, Shanghai, China), and stored in vials for further analysis. In addition, quality control (QC) samples were prepared by mixing equal amounts of each imperial chrysanthemum tea sample to monitor instrument stability and data reproducibility. The order of injection was QC-1, WYHJ-1, WYHJ-2, WYHJ-3, QC-2, JSHJ-1, JSHJ-2, JSHJ-3, QC-3.

#### 2.3.2. UPLC Conditions and ESI-Q TRAP-MS/MS

The non-volatile metabolites in the samples were analyzed using a UPLC-ESI-MS/MS system. The UPLC system utilized was the Sciex ExionLC™ AD (Applied Biosystems, Foster City, CA, USA), with the Agilent SB-C18 column (1.8 µm, 2.1 mm × 100 mm). The tandem mass spectrometry system employed was the Sciex 4500 QTRAP system (Applied Biosystems). The UPLC analytical conditions and the ESI source operation parameters were set as previously described [17].

Substance identification was performed using secondary spectrum information from the commercial Metware Database (MWDB, Wuhan, Hubei Province, China).

### 2.4. Analysis of Volatile Metabolites in Imperial Chrysanthemum Teas

#### 2.4.1. Sample Pretreatment

Imperial chrysanthemum tea samples (500 mg) were accurately weighed into a headspace vials (Agilent Tech., Little Falls, DE, USA), and a saturated sodium chloride solution and 10 µL of the internal standard solution were added (50 μg/mL of [3,4,5-D3]-furfural). This was followed by extraction via fully automated headspace solid phase microextraction (HS-SPME) for GC-MS analysis. QC sample preparation and injection order were the same as LC-MS analysis.

#### 2.4.2. GC-MS/MS Analysis

The GC and MS acquisition condition were set as previously described [17]. Briefly, the analysis was performed using a DB-5MS capillary column (30 m × 0.25 mm × 0.25 μm; Agilent J&W Scientific, Folsom, CA, USA) with high-purity helium (purity not less than 99.999%), at a constant flow rate of 1.2 mL/min, as the carrier gas. The temperature at the inlet was 250 °C. Further, the mass spectrometry conditions were as follows: ion source (EI), electron bombardment; ion source temperature, 230 °C; quadrupole temperature, 150 °C; mass spectrometry interface temperature, 280 °C; and electron energy, 70 eV. The MS was set in the ion monitoring mode (SIM) to enable the identification and quantification of the analytes.

The relative contents of the metabolites were calculated by comparing the peak areas of a given component to those of the internal reference compound. The relative metabolite content was calculated using the Equation (1).

(1) $$C_{i\;}=\;\frac{V_{S}\;\times \;C_{S}}{M}\;\times \;\frac{I_{i}}{I_{s}}\;\times \;10^{-3}$$

where *C_i_* is the relative content of a identified metabolite (μg/g); *V_s_* is the volume of the internal reference compound (μL), *C_s_* is the concentration of internal reference compound (μg/mL), *M* is mass of the sample (g), *I_s_* is the peak area of internal reference compound, and *I_i_* is the peak area of a identified metabolite.

### 2.5. Measurement of Total Flavonoids and Polysaccharide Content

The total flavonoid contents of the imperial chrysanthemum tea samples were determined as previously described [18] with slight modification. Briefly, 1.00 g tea samples were accurately weighed and mixed with 30 mL of 70% methanol and extracted in water at 60 °C for 30 min. Thereafter, centrifugation was performed at 1000*× g* and the resulting supernatant was fixed to 100 mL for further analysis. To prepare the analysis mixture, 2% aluminium trichloride methanol solution (5 mL) was mixed with an equal volume of the sample solution. This was then followed by absorbance measurements at 510 nm using methanol as the blank control. Then, the total flavonoid contents of samples were calculated according to the quercetin standard curve and expressed as milligram equivalent (QE) of quercetin per gram of imperial chrysanthemum tea.

The polysaccharide content was measured using the phenol-sulfuric acid method [6]. Briefly, crushed imperial chrysanthemum teas (0.05 g) was extracted with 1 mL of water at 100 °C for 2 h, centrifuged (1000*× g*, 10 min), and the supernatant was tested. Then, 2 mL of a 5% phenol solution was added to 0.1 mL sample solution and mixed. Subsequently, concentrated sulfuric acid (7 mL) was slowly added, and the mixture was allowed to react in a boiling water bath for 15 min. After cooling to room temperature, the absorbance of the final solution was measured at 490 nm. Polysaccharide content was calculated using glucose as a standard and expressed as milligrams of glucose equivalents (GE) per gram of imperial chrysanthemum tea.

### 2.6. Preparation of Imperial Chrysanthemum Tea Extracts

The two imperial chrysanthemum teas were ground using a grinder (MM 400, Retsch, Germany). Thereafter, the powder samples were extracted with a 70% methanol solution (1:30 of powder: methanol, g/mL) and concentrated to 1/4 of its initial volume using a vacuum rotary evaporator (RE-201D, Dufu Instruments Plant, Zhengzhou, China). The concentrated solution was then placed in a vacuum freeze-drying machine (SCIENTZ-10N, Scientz Biotechnology Co., Ltd., Ningbo, China) for 24 h. Finally, the extract powder was collected and stored at −20 °C until further analysis.

### 2.7. Determination of Antioxidant Capacity

The antioxidant capacity of imperial chrysanthemum teas was evaluated in vitro using free radical scavenging ability towards DPPH, ABTS^+^, OH^−^, and reducing potency test.

The radical scavenging ability of the extracts were determined using an assay kit (Number: DPPHFRS-F48S-N (1620), TAOCA-F48S-N (1620), and HFRS-F48S-N (1620), Enzyme-linked Biotechnology Co., Ltd., Shanghai, China) according to the manufacturer’s instructions. Ascorbic acid (V_C_) was used as a positive control. Inhibitory concentration at 50% (IC_50_) reflects the antioxidant concentration of the tested samples required to neutralize 50% of the initial concentration of free radicals. The inhibitory effects were evaluated at five different concentrations to adequately cover the linear portion of the dose-response curve and accurately determine the IC_50_.

The ferric reducing antioxidant power (FRAP) was determined using an assay kit (Number: TAOCF-W96S-N (1620), Shanghai Enzyme-linked Biotechnology Co., Ltd., Shanghai, China). Ascorbic acid (V_C_) was used as a positive control. FRAP value was expressed in Trolox equivalents per milliliter of the extract (μmol Trolox/mL) at extracts concentration of 100 mg/mL (extracts/buffer solution). Trolox curve was Y = 1.2416X + 0.0134, R^2^ = 0.9996, where X represents the concentration of Trolox (µmol/mL), Y represents absorption value.

### 2.8. Anti-Diabetic Assays In Vitro

#### 2.8.1. α-Amylase Activity Determination

The inhibition of α-amylase activity was assessed as previously described [19]. Briefly, 0.5 mL of 1 U/mL α-amylase solutions (from porcine pancreatic, Adamas, Shanghai, China) were placed in test tubes and 0.5 mL of the sample solution at different concentrations were added. Following incubation in a 37 °C water bath for 15 min, 0.5 mL of 1% soluble starch solution was added to the mixtures. After thorough mixing, the reactions were incubated for a further 10 min at 37 °C. Finally, 0.2 mL of DNS reagent was added to terminate the reaction. The reaction mixture was then boiled in a water bath for 5 min and cooled to room temperature. This was followed by the addition of 7 mL of phosphate buffer solution (0.1 mol/L, pH 6.8) after which, absorbance measurements were performed at 540 nm. The α-amylase inhibition activities of the samples were then calculated according to Equation (2) using acarbose as positive control.

(2) $$\mathrm{Inhibition}\;\left(\%\right)\;=\;(1\;-\;\frac{A_{1\;}-\;A_{2}}{A_{3}\;-\;A_{4}})\;\times \;100$$

where *A*_1_ represents the absorbance of the mixed solution containing the test samples; *A*_2_ represents the absorbance of the mixed solution without the enzyme, but containing the sample and phosphate buffer solution; *A*_3_ represents the absorbance of phosphate buffer solution with the enzyme but without the test samples, and *A*_4_ represents the absorbance of the mixed solution containing the phosphate buffer solution replacing the sample and the enzyme. The data were reported as IC_50_ values for each tested sample.

#### 2.8.2. α-Glucosidase Activity Determination

The inhibition of α-glucosidase activity was estimated as previously described with minor modifications [20]. In short, 0.2 mL of the samples at different concentrations were placed in centrifuge tubes and then 0.2 mL of 0.5 U/mL α-glucosidase solution (from yeast, Yuanye, Shanghai, China) was added. This was followed by thoroughly mixing, after which the mixture was heated in a water bath at 37 °C for 10 min. Next, 0.2 mL of PNPG solution (2.5 mM, pH 6.8) and 0.8 mL of phosphate buffer solution (0.1 mol/L, pH 6.8) were added, and the mixture was incubated at 37 °C for 30 min. Then, to terminate the reaction, 2 mL of 1 mol/L Na_2_CO_3_ was added and the mixed solution was allowed to stand for 30 min. Finally, absorbance measurements were performed at 405 nm using acarbose as the positive control. The inhibitory activities of the different tested samples, calculated according to Equation (2), were reported as IC_50_ values.

### 2.9. Anti-Inflammatory Assays In Vitro

#### 2.9.1. Xanthine Oxidase (XOD) Activity Determination

An assay to measure the ability of the extracts to inhibit uric acid production, and thus, indirectly measure their anti-inflammatory effects, was realized by performing an anti-XOD assay. The extracts were dissolved in 10% DMSO and diluted to the desired concentrations. Thereafter, 20 μL of 0.20 mM phosphate buffer solution buffer (pH 7.5) was mixed with 20 μL of the sample solution and 80 μL of 0.20 U XOD solution (from cow’s milk, Bioss, Beijing, China) in a 96-well plate, followed by incubation for 3 min at 37 °C in an enzyme activity counter. Finally, 0.12 mM xanthine was added to the plate and the absorbance measurements were recorded at 295 nm every 30 s for 6 min [21]. The positive control used was allopurinol. Further, the XOD inhibitory action of extracts were then calculated according to Equation (3).

(3) $$\mathrm{Inhibition}\;(\%)\;=\;\frac{A_{blank}\;-\;A_{sample}}{A_{blank}}\;\times \;100$$

where *A_blank_* represents the absorbance of the mixed solution without the sample extracts and *A_sample_* represents the absorbance of the mixed solution containing the sample extracts. The inhibitory activities of the tested samples were expressed as IC_50_ values.

#### 2.9.2. Lipoxygenase (LOX) Activity Determination

An anti-LOX activity assay for the imperial chrysanthemum tea extracts was performed as previously described [22] with some modifications. Briefly, sample extracts (20 μL), phosphate buffer solution solution (150 μL, 100 mM, pH 7.4), LOX enzyme (20 μL, from soybean, Adamas, Shanghai, China), and linoleic acid (60 μL) were mixed in a 96-well microplate reader. Thereafter, the reaction mixture was incubated at 30 °C for 8 min and absorbance measurements were performed at 234 nm. The positive control was NDGA. Further, the LOX inhibitory activities of the extracts were expressed as IC_50_ values according to Equation (3).

### 2.10. Date Processing and Statistical Analysis

The prcomp function in R (www.r-project.org) was used for unsupervised principal component analysis (PCA), prior to which the data underwent unit variance scaling. Further, differential metabolites were determined using VIP (VIP > 1) and absolute Log_2_FC (|Log_2_FC| ≥ 1.0) values. Multiple comparison correction was performed using the Benjamini-Hochberg (BH) method for false discovery rate (FDR) control. All analyses were performed using unpaired, two-sided *t*-tests, assuming unequal variances, with a confidence level of 0.95. The normalized signal intensities of the metabolites were visualized as color spectra with unit variance scaling using TB tools (Guangzhou, China). All assays were conducted in triplicate, and the results are presented as the means of three replicate tests. Statistical analysis was performed using one-way analysis of variance (ANOVA) in SPSS software (version 22.0, SPSS Inc., Chicago, IL, USA). A post hoc analysis was conducted using Tukey’s b test, with *p* < 0.05 considered statistically significant.

## 3. Results and Discussion

### 3.1. Analysis of Fresh Flower Morphology in Imperial Chrysanthemum Tea

**Figure 1:**
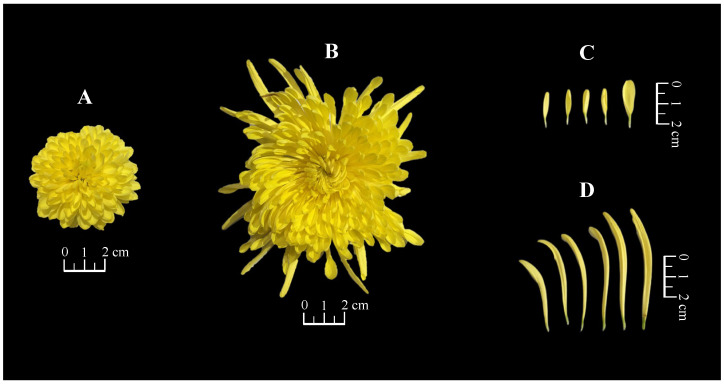
Morphological characteristics of fresh flowers in (**A**) Wuyuan Huangju (WYHJ) and (**B**) Jinsi Huangju (JSHJ), and the ligulate florets of (**C**) WYHJ and (**D**) JSHJ.

The morphological characteristics of the fresh flowers of the two imperial chrysanthemum teas are shown in Figure 1 and Table 1. Significant differences were observed between the two teas with respect to weight, diameter, and ligulate flower length (*p* < 0.01). The weight of JSHJ (8.21 ± 0.89 g) was approximately 4.5-fold that of WYHJ (1.82 ± 0.35 g). Further, the capitula of both WYHJ and JSHJ were identified as the double-petal type, with fewer tubular and mass-ligulate florets [4]. There was no significant difference between the two tea varieties with respect to the number of ligulate florets; however, JSHJ was approximately 3.6-fold longer than WYHJ.

### 3.2. Analysis and Identification of Non-Volatile Metabolites in Imperial Chrysanthemum Teas

#### 3.2.1. Mass Spectrometric Analysis of Non-Volatile Metabolites in Imperial Chrysanthemum Teas

**Figure 2:**
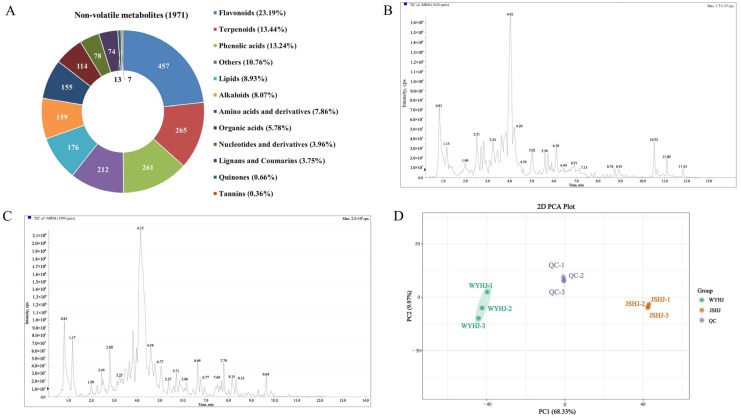
Classes of non-volatile metabolites in imperial chrysanthemum teas obtained through a widely targeted metabolic method (**A**) and the method reliability evaluation (**B**–**D**). (**B**) Overlap analysis of the total ion current diagram for quality control (QC) samples in positive ionization mode. (**C**) Overlap analysis of the total ion current diagram for QC samples in negative ionization mode. (**D**) PCA plot of all test samples.

The two imperial chrysanthemum teas varieties, WYHJ and JSHJ, were investigated via a widely targeted metabolomics approach. Altogether, 1971 non-volatile metabolites were identified, including 457 flavonoids, 265 terpenoids, 261 phenolic acids, 176 lipids, 159 alkaloids, 155 amino acids, 114 organic acids, 78 nucleotides, 74 lignans and coumarins, 13 quinones, 7 tannins, and 212 other metabolites (e.g., saccharides, vitamins, and chromones) (Figure 2A and Table S1). To assess the method’s reliability, we prepared triplicate quality control (QC) samples. As shown in Figure 2B,C, the analysis of the overlap in the total ion current diagram of the QC samples in both the positive and negative ionization modes demonstrated great reproducibility. An unsupervised PCA model was used to monitor the data for both the QC and tested samples, depending on the 1971 non-volatiles obtained in the current research (Figure 2D). The two tea varieties were distinctly separated, and the QC samples were positioned at the centroids of all the specimens tested. This finding confirmed the dependability of the approach and also indicated that the two imperial chrysanthemum tea varieties contain different non-volatile compounds.

#### 3.2.2. Significantly Different Non-Volatile Metabolites Between Two Imperial Chrysanthemum Teas

OPLS-DA was conducted to identify significant differences in non-volatile compounds between the two imperial chrysanthemum tea varieties; VIP ≥ 1.0, FC ≥ 2 or ≤0.5, and *p* < 0.05 indicated significant differences. A total of 744 significantly different non-volatile metabolites were identified, including 250 flavonoids, 122 terpenoids, 83 phenolic acids, 49 alkaloids, 45 amino acids, 42 lipids, 32 organic acids, 29 lignans and coumarins, 23 nucleotides, 7 quinones, and 62 others (Table S2). Among these differential compounds, 474 non-volatile metabolites were upregulated in WYHJ. A more detailed analysis of the differences in non-volatile metabolites between the two imperial chrysanthemum teas is summarized as follows:

(1) Flavonoids

Reported, flavonoids, which possess anti-inflammatory and antioxidant activity, are important metabolites in *C. morifolium* flowers, and their levels in this flower are higher than those of other compounds [13,23,24,25]. However, individual chrysanthemum samples may differ significantly in terms of flavonoid quality and quantity [25,26]. In this study, 250 significantly different flavonoids were identified between the two imperial chrysanthemum tea varieties, including 116 flavones, 75 flavonols, 24 flavanones, 3 flavanonols, 16 isoflavones, 7 chalcones, 3 anthocyanidins, 3 flavanols, and 3 aurones (Table S2).

**Figure 3:**
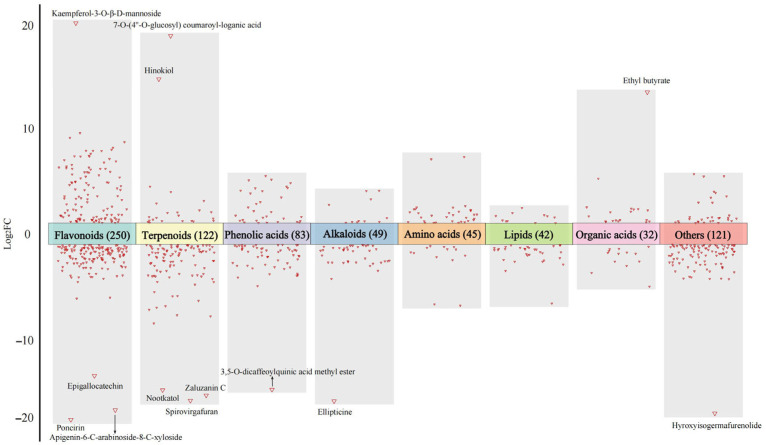
The volcano plot of significantly different non-volatile metabolites in imperial chrysanthemum teas based on their Log_2_FC values.

Flavones are the most abundant flavonoids in *Chrysanthemum morifolium*, followed by flavonols and flavonones [7]. The results of the present study indicated the number of flavones and flavanones upregulated in WYHJ was higher than that in JSHJ (Table S2). Additionally, the volcano plot of different flavonoids is shown in Figure 3. The absolute Log_2_FC values of epigallocatechin, poncirin, apigenin-6-C-arabinoside-8-C-xyloside, and kaempferol-3-O-β-D-mannoside were greater than 10 (|Log_2_FC| ≥ 10), implying that these compounds were characteristic differences in flavonoids between two chrysanthemum varieties. Previous research has shown that flavonoids with significant differences in content among *Chrysanthemum morifolium* cultivars, e.g., apigenin glycosides, diosmetin glycosides, and acacetin glycosides, can be used as indicators for quantitative identification [27].

(2) Terpenoids

Terpenoids are important bioactive components of *Chrysanthemum* that have demonstrated anti-inflammatory, antibacterial, and anti-viral properties [28], and based on their chemical structure, they are classified under four categories, namely, monoterpenes, sesquiterpenoids, diterpenoids, and triterpenes. Further, they are primarily present in essential oils, as identified via GC-MS analysis [29]. It has also been reported that most of non-volatile terpenoids are purified from aqueous ethanolic *Chrysanthemum* extracts using various adsorbents, including silica gel, Sephadex LH-20, or via C18 reversed phase and semi-preparative HPLC [28]. Using updated detection methods, 265 terpenoids were identified via UPLC-MS/MS, of which 122, including 16 monoterpenes, 76 sesquiterpenoids, 11 diterpenoids, and 19 triterpenes, were significantly different between the two imperial chrysanthemum teas.

The terpenoids content, particularly, sesquiterpenoids content, was significantly higher in WYHJ than in JSHJ (Table S2). Seventy out of the 79 differential sesquiterpenes were upregulated in WYHJ. Reportedly, sesquiterpenoids, a class of C15 metabolites that are biosynthetically derived from isoprenoid precursors, are widely distributed in *Chrysanthemum,* and their skeletons have been elucidated in many members of the *Chrysanthemum* genus [12,30]. Consistent with previously reported findings, higher levels of spathulenol, matricarin, and β-dictyopterol in WYHJ have also been observed in *Chrysanthemum indicum* [12] and *Chrysanthemum morifolium* [28], respectively. According to their Log_2_FC values, zaluzanin C, nootkatol, spirovirgafuran, hinokiol, and 7-O-(4″-O-glucosyl) coumaroyl-loganic acid were characteristic differences in terpenoids (Figure 3).

(3) Phenolic acids

Phenolic acids are the main active constituents of *C. morifolium*, and the derivatives of the phenolic acids, chlorogenic and caffeoylquinic acids, exert antibacterial, anti-viral, anti-infective, and anti-inflammatory properties [7,31]. Further, phenolic acids, such as hydroxycinnamic and hydroxybenzoic acids, are typically found conjugated with other polyphenols, quinic acid or glucose; they rarely exist in the free form [32]. Caffeoylquinic acid, which is present in *Chrysanthemum morifolium* and is formed via the combination of caffeic acid (hydroxycinnamic acid) and quinic acid, has been extensively investigated [33]. The chromatographic fingerprints of 30 *Chrysanthemum morifolium* flowers revealed that 3,5-O-dicaffeoylquinic acid, chlorogenic acid, and 4,5-O-dicaffeoylquinic acid could be used as markers for quality evaluation [31]. The levels of these three quality marker components were not significantly different between the two imperial chrysanthemum teas investigated in this study (Table S1), indicating that their quality evaluations at the phenolic acid level were similar.

The changes in the 83 differential phenolic acids between the two imperial chrysanthemum teas are shown in Figure 3 and Table S1. Thirty-one of the 39 differential hydroxycinnamic acids, including caffeic acid, caffeoylquinic acid esters, and caffeoyl glycoside quinic acids, were upregulated in WYHJ. In addition, 3,5-O-dicaffeoylquinic acid methyl ester was recognized as characteristic differences in phenolic acids by Log_2_FC values. However, the level of differential hydroxybenzoic acids, including methyl gallate, 2,3-dihydroxybenzoic acid, and protocatechuic acid, was similar between the two imperial chrysanthemum teas.

(4) Alkaloids

One hundred and fifty-nine alkaloids were identified, of which 49 were differential alkaloids between WYHJ and JSHJ (Tables S1 and S2). Alkaloids are important molecules in *Chrysanthemum*, and their accumulation is greatly affected by the environment [34]. The number of upregulated alkaloids in WYHJ was significantly higher than that in JSHJ, and the level of ellipticine was the greatest difference between them (Figure 3).

(5) Others

In addition to the differences in the abovementioned non-volatile metabolites, many other categories of metabolites, including amino acids, lipids, organic acids, nucleotides, lignans, coumarins, saccharides, and vitamins, also showed significant differences between the two imperial chrysanthemum teas (Figure 3, Table S2).

A total of 155 amino acids were identified, of which 45 were differential metabolites. Amino acids, which are nutrients required by the humans, are important flavor components in *Chrysanthemum* tea [35]. Further, eight essential amino acids were identified in the two imperial chrysanthemum tea varieties, and of these, methionine, valine, and lysine were significantly abundant in JSHJ. Additionally, the levels of 32 amino acids in JSHJ, including asparagine and glutamine, which are abundant in *Chrysanthemum dichrum* leaves, flower buds, and blooming [36], were significantly higher than those in WYHJ.

Forty-two differential lipids were identified in WYHJ and JSHJ, including 26 free fatty acids, seven glycerol esters, four lysophosphatidyl cholines (LPC), four lysophosphatidyl ethanolamines (LPE), and one sphingolipid. Fatty acids, particularly unsaturated fatty acids in *C. morifolium*, exert significant anti-inflammatory effects [37]. Twenty-three out of the 26 differentially expressed free fatty acids were upregulated in WYHJ.

Thirty-three differential organic acids were identified between the two imperial chrysanthemum teas, and of these, 20 were upregulated in WYHJ. Specifically, ethyl isobutyrate and ethyl butyrate were identified as the top two upregulated non-volatile metabolites based on their Log_2_FC values (Figure S1). Similar studies on *C. mongolicum* and *C. rhombifolium* have shown that the levels of organic acids and their derivatives are significantly higher in *C. rhombifolium* and these potential biomarkers (FC > 7 or <−7) have considerable practical value for taxonomic classification [38].

Nucleotides and their derivatives are essential nutritive and functional compounds in *C. mongolicum* [39]. Five nucleotides have been identified as quality defining markers for nine different *C. mongolicum* tea varieties of different origins [40]. In the current study, 23 nucleotides and their derivatives were identified as differential metabolites, and among these, 22 showed higher levels in WYHJ than in JSHJ. Coumarins, lignans, quinones, saccharides, vitamins, ketones, chromones, lactones, aldehydes, and tannins showed varying degrees of differences between the two imperial chrysanthemum teas. The levels of coumarins, saccharides, vitamins, and actones, including antioxidant vitamin C [41] and anti-obesity cichoriin [42], were significantly higher in WYHJ than in JSHJ.

### 3.3. Analysis and Identification of Volatile Metabolites in Imperial Chrysanthemum Teas

#### 3.3.1. Volatile Metabolite Profiles in Imperial Chrysanthemum Teas

**Figure 4:**
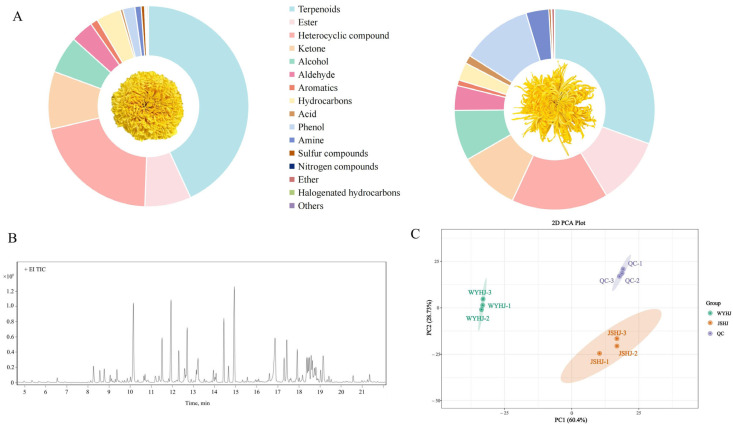
(**A**) Classes of volatile metabolites in imperial chrysanthemum teas. (**B**) Overlap analysis of the total ion current diagram for quality control (QC) samples. (**C**) Principal component analysis (PCA) plot of all test samples.

In this study, we identified 1039 volatiles across 16 categories in the two imperial chrysanthemum teas (Figure 4A and Table S3), with terpenoids showing predominance in this regard, accounting for 43.10 and 30.55% of the total volatile metabolites in WYHJ and JSHJ, respectively. Further, the composition of heterocyclic (20.74 and 15.58%), esters (7.47 and 10.79%), ketones (9.30 and 9.80%), alcohols (6.01 and 8.13%), aldehydes (3.65 and 3.98%), aromatics (1.19 and 0.92%), hydrocarbons (3.98 and 2.90%), acids (0.41 and 1.32%), amines (1.00 and 3.68%), sulfur compounds (0.55% and 0.34%), nitrogen compounds (0.20 and 0.12%), ethers (0.15 and 0.43%), halogenated hydrocarbons (0.23 and 0.02%), and others (0.03 and 0.01%) were relatively similar in both WYHJ and JSHJ. Notably, the proportion of phenols was significantly different, 1.97 and 11.42% in WYHJ and JSHJ, respectively.

QC samples with three replicates were prepared to evaluate the reliability of the method. An overlay plot of the total ion flow chart (TIC chart) for the mass spectrometry detection of QC samples is shown in Figure 4B. The results obtained indicated that the overlap of the total ion current curves for metabolite detection was high, indicating consistent retention times and peak intensities. This observation implied that the mass spectrometry analysis demonstrated excellent signal stability in the analysis of same sample at multiple time points, ensuring the replicability and dependability of the data. Similarly, the PCA plot (Figure 4C) showed a clear separation trend among the samples, implying the presence of metabolite differences within the sample groups.

#### 3.3.2. Significantly Different Volatile Metabolites Between the Two Imperial Chrysanthemum Teas

**Figure 5:**
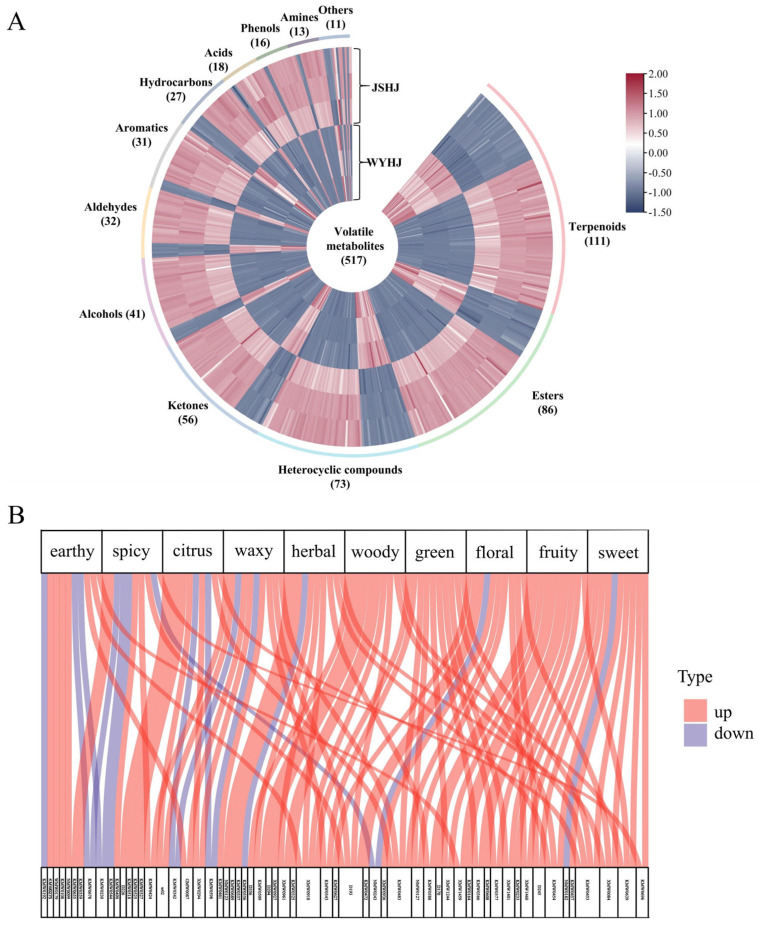
(**A**) Heatmap and (**B**) flavor sankey of significantly different volatile metabolites in imperial chrysanthemum teas. WYHJ: *Imperial chrysanthemum* ‘Wuyuan Huangju’, JSHJ: *Imperial chrysanthemum* ‘Jinsi Huangju’.

Using the same identification method as was used for the identification of differential non-volatiles, 517 differential volatiles, including 111 terpenoids, 86 esters, 73 heterocyclic compounds, 56 ketones, 41 alcohols, 32 aldehydes, 31 aromatics, 27 hydrocarbons, 18 acids, 16 phenols, 13 amines, and 11 others, were identified (Figure 5A and Table S4). In total, 145 and 372 volatile metabolites were upregulated in WYHJ and JSHJ, respectively.

Terpenoids, some of which possess anti-inflammatory, anti-viral, anti-tumor, osteoporosis treatment, and anti-aging effects, are the primary volatile compounds in *Chrysanthemum* varieties and their wild relatives [7]. In this study, 111 of the 239 identified terpenoids were present in the two imperial chrysanthemum teas. More terpenoids, including verbenone, eucalyptol, ylangene, α-pinene, and β-phellandrene, which have been previously reported as the primary volatile compounds in *Chrysanthemum*, showed higher levels in JSHJ than in WYHJ [43]. Additionally, to better compare the different volatile metabolites in WYHJ and JSHJ, bar charts of the top 20 metabolites were generated based on the obtained Log_2_FC values (Figure S2). Particularly, six terpenoids, namely, cis-chrysanthenol, linalyl acetate, trans-carvone oxide, carvenone, p-mentha-1(7),2-dien-8-ol, and 1-methyl-4-(1-methylethyl)-3-cyclohexen-1-ol, were among the top 20 differential metabolites and were upregulated in JSHJ. Conversely, only α-cadinene belonging to the top 20 differential metabolites was upregulated in WYHJ.

Except for sulfur compounds, the numbers of upregulated esters, heterocyclic compounds, ketones, alcohols, aldehydes, aromatics, hydrocarbons, acids, phenols, and amines in JSHJ were higher than those in WYHJ. Further, among the 13 differential metabolites in the top 20 metabolites, 12, except for the terpenoids, showed an upward trend in JSHJ (Figure S2). These included trans-chrysanthenyl acetate (esters), 3-methylheptyl acetate (esters), 2-methyl-1,3-dithiacyclopentane (heterocyclic compounds), 1-(furan-2-yl)-2-methylpentan-1-one (heterocyclic compounds), acetophenone (ketones), 3-methyl-1,2-benzenediol (alcohols), β-4-dimethyl-3-cyclohexene-1-ethanol (alcohols), isocyclocitral (aldehydes), 2-nonenal (aldehydes), 4-(1-methylethenyl)-1-cyclohexene-1-carboxaldehyde (aldehydes), 4-heptyl-phenol (phenols), and memantine (amine).

Overall, the levels of volatile metabolites were higher in JSHJ than in WYHJ. This phenomenon is not only related to factors, such as different *Chrysanthemum* tea subtypes [14], harvest time, origin, and processing methods [7], but also to the size of the capitulum of the plant. A previous study showed that larger capitulae tend to emit more volatile compounds, particularly terpenoids, than their smaller counterparts [16].

#### 3.3.3. Differential of Potential Volatile Flavor Metabolites in the Two Imperial Chrysanthemum Teas

The volatile composition of chrysanthemum tea plays a crucial role in determining its flavour and overall evaluation [14]. To further compare the differences in flavor metabolites between WYHJ and JSHJ, we annotated the odor of the identified volatile metabolites based on relevant websites (http://www.thegoodscentscompany.com or http://perflavory.com/ or http://www.odour.org.uk/odour/index.html or http://foodflavorlab.cn, accessed on 25 March 2024) and the literature [44]. Thus, a total of 502 volatile metabolites with odor characteristics were annotated (Table S3), and the top 10 odor flavor characteristics were selected from the radar chart (Figure S3). Of the top 10 flavor characteristics, the predominant ones included sweet (80), fruity (59), floral (52), green (50), woody (38), herbal (32), waxy (25), citrus (24), spicy (20), and earthy (19). Similar sensory odors (floral, woody, grassy, fruity, sour, and minty) have been reported for *Chrysanthemum* essential oils [45].

For those aforementioned 10 odors, flavor Sankey were drawn with differential volatile metabolites by top 10 value of VIP (Figure 5B). In this diagram, an upward trend (red) indicated a higher content for a given metabolite in JSHJ, whereas blue indicated an upregulation trend in WYHJ. These differential flavor metabolites were mainly upregulated in JSHJ, particularly concerning the odors of fruit, green, and woody. Additionally, linalyl acetate, undecanal, p-mentha-1,8-dien-7-ol, and isocyclocitral exhibited various odors in the differential flavor regulation between the two imperial chrysanthemum teas. However, the flavor identification of these metabolites was only based on relevant websites, these association studies remain speculative in the absence of sensory validation. Future research should prioritize an integrated approach to unequivocally decipher the flavor chemistry of imperial chrysanthemum teas.

### 3.4. Antioxidant Activity

Chrysanthemum tea had been documented for antioxidant function, including the ability to scavenge free radicals in which an excessive amount can initiate radical chain reactions with proteins, lipids, DNA, and other biomolecules, and then cause direct damage to human tissues [46]. Three different free radicals including DPPH, ABTS^+^, and OH^−^ were chosen to estimate the radical scavenging capacity of the two imperial chrysanthemum teas. Furthermore, FRAP was evaluated for reducing potency in this study.

Total flavonoids content (TFC), total polysaccharides content (TPC), reducing potency, and IC_50_ values of those radical scavenging abilities were presented in Table 2. TFC in WYHJ (96.43 ± 1.63 mg QE/g) were significantly higher than JSHJ (73.16 ± 2.04 mg QE/g), while TPC in WYHJ and JSHJ had no significant difference with 43.96 ± 0.32 and 43.94 ± 1.17 mg GE/g, respectively (*p* < 0.05). Same comparison difference as TFC in the two imperial chrysanthemum teas, WYHJ extract exhibited the stronger DPPH, ABTS^+^, and OH^−^ radicals scavenging activity, as the IC_50_ values were 1.04 ± 0.01 mg/mL, 1.27 ± 0.04 mg/mL, and 4.58 ± 0.07 mg/mL. FRAP results showed a similar trend as free radical scavenging activity; the stronger FRAP were 73.99 ± 2.56 µmol Trolox/mL in WYHJ. Compared to antioxidant V_C_, the imperial chrysanthemum teas had weaker anti-free radical and reducing abilities.

Antioxidant activity is the synergistic effect of multiple antioxidant components and is higher than the superposition effect of individual antioxidant components [19]. WYHJ exhibited the stronger antioxidant activity, which is associated with its higher upregulated level of non-volatile metabolite monitored by widely targeted metabolomics (Figure 2A).

### 3.5. α-Amylase and α-Glucosidase Inhibitory Effects

A strong relationship between postprandial hyperglycemia and type 2 diabetes mellitus has been demonstrated [47]. Further, the inhibition of α-amylase and α-glucosidase represents a potential treatment strategy for post-meal hyperglycemia based on retarding carbohydrate metabolism and reducing glucose resorption. Thus, contributes to the management of type 2 diabetes mellitus and its associated conditions [48]. Flavonoids have been regarded as a suitable resource for further research on α-amylase and α-glucosidase inhibitors [11,49]. In total, 457 flavonoids were identified in the two imperial chrysanthemum teas by widely targeted metabolomics (Table S1); however, the efficacies of imperial chrysanthemum teas in inhibiting α-amylase and α-glucosidase activities were unclear. Table 3 shows the IC_50_ values of extracts from WYHJ and JSHJ with respect to the inhibition of α-amylase and α-glucosidase activities. WYHJ exhibited stronger α-amylase inhibitory potency (IC_50_ of 1403.63 ± 166.49 μg/mL) than JSHJ (IC_50_ of 1521.48 ± 20.42 μg/mL), but was weaker than acarbose in this regard (IC_50_ of 166.43 ± 19.71 μg/mL). Interestingly, the α-glucosidase inhibitory activity of WYHJ was stronger than those of acarbose and JSHJ (IC_50_ of 307.94 ± 37.32 μg/mL).

Flavonoids may inhibit α-amylase and α-glucosidase by binding to amino acid moieties close to the action point of the enzyme via electrostatic interactions and hydrogen bonds, leading to alterations in enzyme conformation and the ultimate prevention of the attachment of substrate-bound enzymes [50]. In Table 3, the TFC in WYHJ (96.43 ± 1.63 mg QE/g) was significantly higher than that in JSHJ (73.16 ± 2.04 mg QE/g). Furthermore, flavonoids (luteolin, acacetin, and buddleoside) were investigated for their inhibitory effects on α-amylase in *Chrysanthemum indicum*, with the inhibition stronger for luteolin than acacetin and weakest for buddleoside based on the binding affinity of the surface plasmon resonance [11]. It is also worth noting that luteolin (>98% by HPLC) has a greater inhibitory effect on α-glucosidase than acarbose, with 3.65 ± 0.77 and 563.60 ± 40.49 μmol/L of IC_50_ values, respectively [51]. Meanwhile, molecular docking simulations confirmed that taxifolin exhibits inhibitory activity against α-glucosidase and α-amylase by forming multiple secondary interactions, such as hydrogen bonds and *π*–*π* stacking [52]. Coincidentally, WYHJ extracts were found to contain higher concentrations of luteolin and taxifolin than JSHJ extracts (Table S2). Therefore, this finding confirmed our hypothesis regarding the partial functionality of imperial chrysanthemum teas, and indicated that their α-amylase and α-glucosidase inhibitory effects resulted from the synergistic effects of multiple flavonoids.

### 3.6. XOD and LOX Inhibitory Effects

Anti-inflammatory and immunomodulatory effects are the most commonly reported traditional efficacy of *Chrysanthemum* [7]. Gout is a type of arthritis that causes inflammation, and hyperuricemia, a condition characterized by the excessive accunulation of uric acid, is a major risk factor for gout. This excessive uric acid production is caused by the oxidation of hypoxanthine and xanthine to uric acid by XOD [9]. Allopurinol inhibits the last stage of the production of uric acid and is currently used as an XOD inhibitor in the treatment of gout. Even though the abilities of the imperial chrysanthemum teas to inhibit XOD were not directly comparable to that of allopurinol, our results indicated that imperial chrysanthemum teas show XOD inhibitory effects, with WYHJ (IC_50_ of 818.51 ± 43.26 μg/mL) showing a higher inhibitory effect than JSHJ (IC_50_ of 1441.66 ± 62.51 μg/mL) in this regard (Table 3). This finding is consistent with previously reported observations; the inhibitory activity of XOD could be primarily attributed to phenolics and flavonoids and is positively correlated with DPPH radical scavenging activity [9,53]. Based on an investigation of 31 batches of C. morifolium ‘Boju’ regarding xanthine oxidase inhibitory activity, Peng et al. [54] found that samples with a higher abundance of characteristic monoterpenoids and flavonoid aglycones exhibited significantly stronger XOD inhibition. Their spectrum–effect relationship analysis further identified three key flavonoids—luteolin, apigenin, and acacetin—as direct XOD inhibitors, along with four monoterpenoids (filifolone, α-terpineol, thymol, and piperitenone) that are closely associated with the observed inhibitory effects.

LOX is produced in humans and plays an essential role in stimulating inflammatory responses [10]. The inhibitory effects of the two imperial chrysanthemum teas and nordihydroguaiaretic acid (the positive control) are shown in Table 3. WYHJ exhibited a superior LOX inhibitory effect compared to JSHJ; however, its inhibitory effect fell short to that of nordihydroguaiaretic acid. The IC_50_ values of WYHJ and JSHJ were 533.78 ± 30.12 and 1184.11 ± 80.63 μg/mL, respectively. Variations in natural metabolites result in differences in LOX inhibitory effects; this phenomenon has been observed in the inflorescences of *Chrysanthemum indicum* and *Tagetes erecta* [55]. Although the in vitro activity of the two imperial chrysanthemum teas was observed in this study, it is important to recognize the limitations of this study. While our findings demonstrate potent inhibitory effects on α-amylase, α-glucosidase, XOD, and LOX in cell-free systems, these results cannot be directly extrapolated to a living organism. Factors such as bioavailability, absorption, metabolism, tissue distribution, and potential systemic effects remain unknown. Therefore, the physiological relevance and therapeutic potential of these imperial chrysanthemum teas require further validation through animal models and ultimately, human clinical trials.

## 4. Conclusions

In this study, we investigated the bioactive metabolites in two imperial chrysanthemum tea varieties, WYHJ and JSHJ, from Chinese medicinal herbal plants and also explored their bioactivities in vitro. Thus, a total of 1971 non-volatile and 1039 volatile metabolites were identified in both WYHJ and JSHJ based on a widely targeted metabolomics approach. Further analysis revealed that WYHJ contained higher levels of non-volatile metabolites (flavonoids, terpenes, phenolic acids), whereas JSHJ displayed elevated levels of volatile metabolites (esters, ketones, alcohols, aldehydes). Phytometabolites play a crucial role in the evaluation of the physiological activity and flavor of plants. WYHJ contained higher levels of bioactive non-volatile substances, such as flavonoids, terpenes, and phenolic acids, which contributed to its stronger physiological activity. Additionally, WYHJ demonstrated potent inhibitory effect on the activities of both α-amylase and α-glucosidase, which are key enzymes associated with hyperglycemia. It also exhibited notable inhibitory effects on the activities of XOD and LOX, both of which are involved in inflammation. Conversely, JSHJ exhibited a greater proportion and number of flavor volatiles. It contained higher amounts of sweet, fruity, and floral substances, possibly owing to its morphological characteristics, such as its larger capitulum size. This study provides novel insights into the comprehensive development and utilization of traditional medicinal plant resources. However, further studies, including in vivo studies, are necessary to validate the hypoglycemic and anti-inflammatory potential of the imperial chrysanthemum teas and to elucidate the associated underlying mechanisms of action. Further, to the metabolomic methodology, our metabolite identifications remain putative (MSI Level 2) due to the lack of confirmation with authentic standards. While we applied high-stringency filters for mass accuracy and MS/MS matching to maximize confidence, future work should aim to definitively confirm the identity of the key metabolites identified here using commercially available reference standards. Nonetheless, our untargeted approach successfully identifies robust metabolic perturbations associated with imperial chrysanthemum teas, providing a strong foundation for these future targeted analyses.

## Figures and Tables

**Table 1:** Morphological characteristics of fresh flowers in imperial chrysanthemum teas.

Species	Weight (g)	Diameter (cm)	Length of Ligulate Florets (cm)	Number of Ligulate Florets
WYHJ	1.82 ± 0.35	5.49 ± 0.24	1.57 ± 0.33	262 ± 10
JSHJ	8.21 ± 0.89 **	11.02 ± 0.45 **	5.73 ± 0.57 **	247 ± 12

Data are presented as mean ± SD (*n* = 3). Statistical significance was determined by single-factor analysis of ANOVA followed by Tukey’s b test. ** denote statistically significant differences among species at *p* < 0.01. WYHJ: *Chrysanthemum morifolium* cv. ‘Wuyuan Huangju’, JSHJ: *Chrysanthemum morifolium* cv. ‘Jinsi Huangju’.

**Table 2:** Comparison of TFC, TPC, and antioxidant activities in vitro of imperial chrysanthemum teas.

Sample	TFC (mg QE/g)	TPC (mg GE/g)	Radical Scavenging Activities (IC_50_)			Reducing Power Assay
			DPPH·(mg/mL)	ABTS^+^ (mg/mL)	OH^−^ (mg/mL)	FRAP (µmol Trolox/mL)
WYHJ	96.43 ± 1.63 **	43.96 ± 0.32	1.04 ± 0.01 ^b^	1.27 ± 0.04 ^b^	4.58 ± 0.07 ^b^	73.99 ± 2.56 ^b^
JSHHJ	73.16 ± 2.04	43.94 ± 1.17	1.96 ± 0.15 ^c^	2.18 ± 0.02 ^c^	6.98 ± 0.08 ^c^	39.18 ± 1.79 ^c^
V_C_	-	-	0.09 ± 0.01 ^a^	0.18 ± 0.02 ^a^	2.68 ± 0.41 ^a^	620.91 ± 8.38 ^a^

WYHJ: *Chrysanthemum morifolium* cv. ‘Wuyuan Huangju’, JSHJ: *Chrysanthemum morifolium* cv. ‘Jinsi Huangju’; TFC: total flavonoids content; TPC: total polysaccharides content; QE: quercetin equivalents; GE: glucose equivalent; V_C_: ascorbic acid; IC_50_ value is the inhibitory concentration of samples required to scavenge 50% of the radicals. Data are presented as mean ± SD (*n* = 3). Statistical significance was determined by single-factor analysis of ANOVA followed by Tukey’s b test. ** denote statistically significant differences among species at *p* < 0.01. Different lowercase letters represented significantly different at *p* < 0.05.

**Table 3:** Comparison of anti-hyperglycemia and anti-inflammatory in vitro of imperial chrysanthemum teas.

Sample	Anti-Hyperglycemia Assays (IC_50_, μg/mL)		Anti-Inflammatory Assays (IC_50_, μg/mL)	
	Anti-α-Amylase	Anti-α-Glucosidase	Anti-XOD	Anti-LOX
WYHJ	1403.63 ± 166.49 ^b^	307.94 ± 37.32 ^a^	818.51 ± 43.26 ^b^	533.78 ± 30.12 ^b^
JSHJ	1521.48 ± 20.42 ^c^	858.58 ± 92.18 ^c^	1441.66 ± 62.51 ^c^	1184.11 ± 80.63 ^c^
acarbose	166.43 ± 19.71 ^a^	654.35 ± 54.36 ^b^	-	-
allopurinol	-	-	30.19 ± 7.48 ^a^	-
NDGA	-	-	-	48.63 ± 9.37 ^a^

WYHJ: *Chrysanthemum morifolium* cv. ‘Wuyuan Huangju’, JSHJ: *Chrysanthemum morifolium* cv. ‘Jinsi Huangju’; XOD: Xanthine oxidase; LOX: Lipoxygenase; NDGA: nordihydroguaiaretic acid; IC_50_ value is the inhibitory concentration of samples required to scavenge 50% of the radicals. Data are presented as mean ± SD (*n* = 3). Statistical significance was determined by single-factor analysis of ANOVA followed by Tukey’s b test. Different lowercase letters represented significantly different at *p* < 0.05.

## Data Availability

The data that support the findings of this study are available from the corresponding author upon reasonable request.

## Supplementary Materials

The following supporting information can be downloaded at: https://www.mdpi.com/article/10.3390/foods14173142/s1, Figure S1. The top 20 differential non-volatile metabolites in WYHJ and JSHJ identified based on their Log_2_FC values. Figure S2. The top 20 differential volatile metabolites in WYHJ and JSHJ identified based on their Log_2_FC values. Figure S3. Flavor radar chart of the top 10 odor flavors with the highest number of annotated metabolites. Table S1. Non-volatile metabolites identified using a widely targeted metabolomic approach in the two imperial chrysanthemum teas. Table S2. Non-volatile differential metabolites in the two imperial chrysanthemum teas. Table S3. Volatile metabolites identified using a widely targeted metabolomic approach in the two imperial chrysanthemum teas. Table S4. Volatile differential metabolites in the two imperial chrysanthemum teas. Table S5. Detailed information of antioxidant and enzyme inhibition experiments of imperial chrysanthemum tea.

## Abbreviations

GC-MS/MS, gas chromatography-tandem mass spectrometry; JSHJ, *Imperial chrysanthemum* var. Jinsi Huangju; LOX, lipoxygenase; PCA, principal component analysis; TFC, total flavonoids content; UPLC-ESI-MS, ultra-performance liquid chromatography-electron spray ionization-mass spectrometry; WYHJ, *Imperial chrysanthemum* var. Wuyuan Huangju; XOD, xanthine oxidase.
