# Supplementary material for: Widely Targeted Metabolomic Analysis of Two Chinese Traditional Herbal Imperial Chrysanthemum Teas and In Vitro Evaluation of Their Hyperglycemia and Inflammation Enzyme Inhibitory Activities
Source: Foods. 2025 Sep 8;14(17):3142. doi: 10.3390/foods14173142 (PMC12427848; doi:10.3390/foods14173142)
Supplement: Supplementary file 1 [file foods-14-03142-s001.zip › Supplementary Figures.pdf]

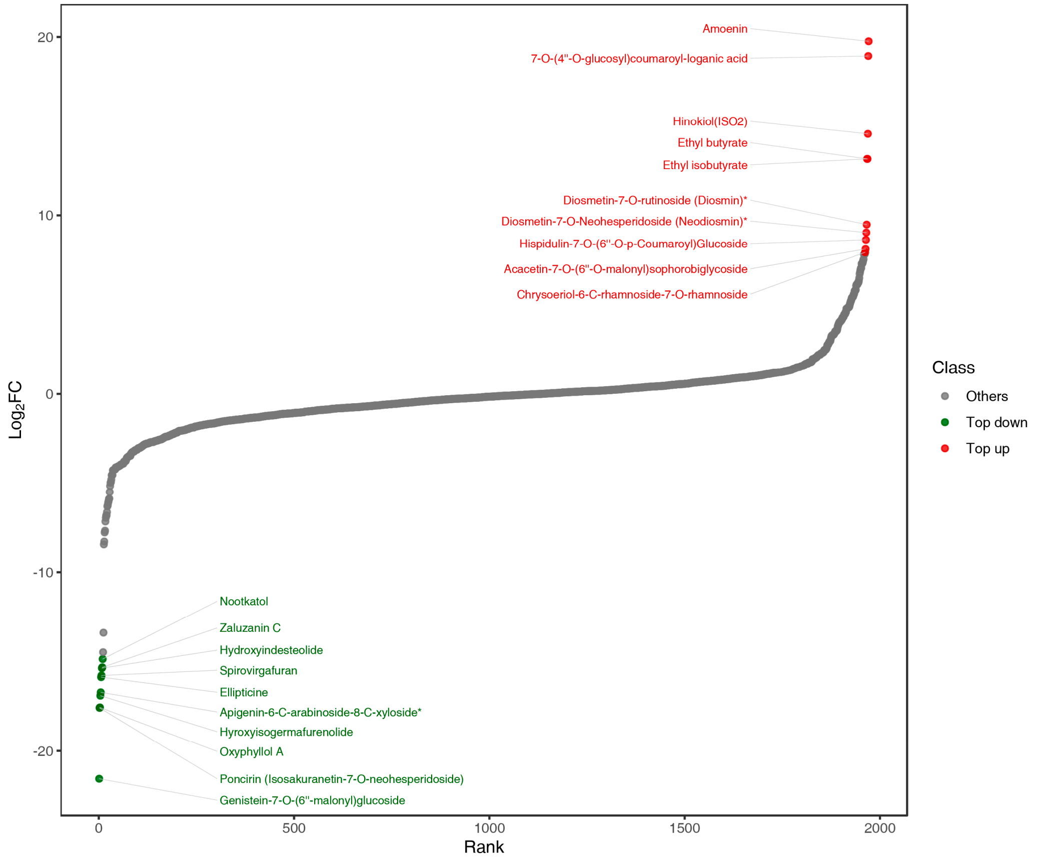


**Figure S1.** The top 20 differential non-volatile metabolites in WYHJ and JSHJ identified based on their $Log_2FC$ values.

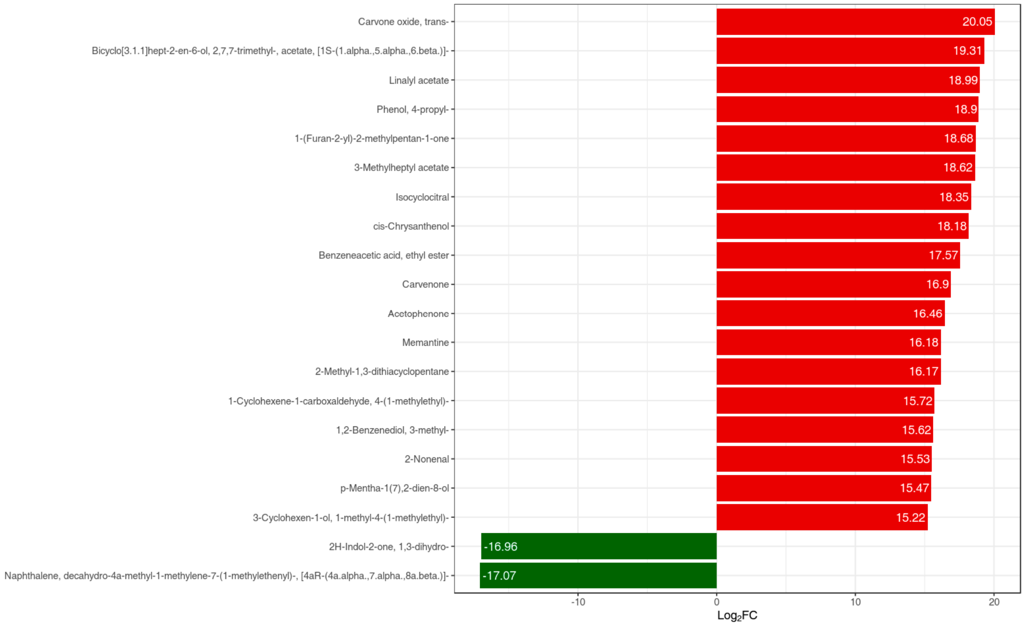


**Figure S2.** The top 20 differential volatile metabolites in WYHJ and JSHJ identified based on their $Log_2FC$ values.

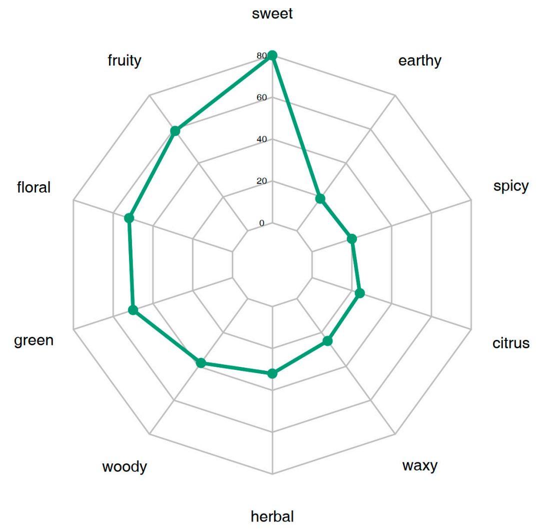


**Figure S3**. The flavor radar chart of top 10 odor flavors with the highest number of annotated metabolites.
